# A Dual-Branch Deep Learning Framework with Explainability for Dental Caries Classification Using Intra-Oral Photographs and Radiographs

**DOI:** 10.3390/jimaging12050207

**Published:** 2026-05-12

**Authors:** Lijuan Ren, Jinjing Chen

**Affiliations:** 1 School of Software Engineering, Chengdu University of Information Technology, Chengdu 610225, China; jinjingchan@foxmail.com; 2 Sichuan Province Engineering Technology Research Center of Support Software of Informatization Application, Chengdu 610225, China

**Keywords:** attention mechanism, data augmentation, deep learning, dental caries detection, explainable AI, medical image analysis

## Abstract

The accurate detection of dental caries is often hindered by modality-specific imaging challenges, such as illumination artifacts in intra-oral photographs and low lesion contrast in radiographs. This study proposes a comprehensive framework comprising three key components: (1) HybridAugment+, an entropy-guided adaptive augmentation strategy that applies stronger transformations to low-information images; (2) DBAttNet, a dual-branch attention network featuring illumination–reflection aware attention (IRAA) for photographs and contrast–frequency-aware attention (CFA) for radiographs; and (3) a CAM-based explainability method, selected through a systematic evaluation of five advanced techniques. This study utilized two datasets derived from public sources, comprising 639 intra-oral photographs (481 caries, 158 healthy) and 456 radiographs (268 caries, 188 healthy). These were annotated by two dentists, with established inter-rater reliability (κ = 0.82 for photographs, κ = 0.79 for radiographs). The experimental results demonstrate that HybridAugment+ improved performance over conventional augmentation by up to 8.72% on photographs and 7.67% on radiographs. Furthermore, DBAttNet achieved F1-scores of 97.90% on photographs and 95.72% on radiographs, outperforming ResNet50, InceptionV3, MSDNet, DCANet, and ARM-Net. A comparative evaluation identified XGrad-CAM as the most suitable explainability method, with optimal visualization thresholds of 30% for photographs and 20% for radiographs. Generalization experiments on ophthalmology (APTOS 2019, Messidor-2) and chest radiography datasets (Kermany CXR, NIH ChestX-ray14) demonstrated consistent performance gains over domain-specific methods (DT-Net, ConvNeXt-Tiny). These results confirm that the core design principles effectively transfer to other modalities facing analogous imaging challenges.

## 1. Introduction

Dental caries, commonly known as tooth decay, remains one of the most prevalent chronic diseases worldwide, affecting approximately 2.5 billion people [1]. The disease process begins with the demineralization of tooth enamel caused by acids produced by oral bacteria, initially manifesting as subtle white-spot lesions on vulnerable tooth surfaces, including occlusal fissures and interproximal spaces [2]. If left untreated, these early lesions can progress to cavitation, pulp involvement, and, ultimately, tooth loss. Early detection is therefore critical in enabling minimally invasive interventions and preventing disease progression [3].

The clinical diagnosis of early caries typically relies on two complementary imaging modalities: intra-oral photographs for the visual inspection of surface changes and bitewing or periapical radiographs for the assessment of subsurface demineralization [4]. However, both modalities present inherent challenges in the identification of early lesions. Intra-oral photographs are susceptible to illumination variations, specular reflections from saliva, and shadows from anatomical structures, all of which can obscure or mimic early demineralization zones [5]. Radiographs, while valuable for detecting interproximal lesions, offer limited contrast between healthy enamel and early-stage demineralization. Their interpretation is further complicated by anatomical noise from overlapping tooth structures [6]. These modality-specific limitations contribute to diagnostic variability, with the reported inter-examiner agreement ranging from moderate to substantial, even among experienced clinicians [7].

Recent advanced approaches in deep learning, particularly convolutional neural networks (CNNs), have shown promise in automating caries detection and reducing diagnostic variability [8,9,10]. Studies have demonstrated the feasibility of transfer learning using architectures such as AlexNet [11], fully convolutional networks (FCNs) [12], and MobileNetV2 [13], achieving accuracy rates between 80% and 96% on various dental image datasets. More recent architectures incorporating attention mechanisms and multi-scale feature extraction have further improved performance [14,15,16]. Despite these advances, current deep learning approaches for caries detection face three fundamental limitations that constrain their clinical utility.

First, robustness to modality-specific artifacts remains insufficient. Most existing methods are designed for, and evaluated on, a single imaging modality—typically either photographs or radiographs, but not both [12,17]. When applied across modalities, these models often fail to generalize due to their distinct noise distributions: illumination artifacts in photographs versus low contrast and anatomical noise in radiographs. Even within a single modality, models trained on curated datasets often degrade when deployed in clinical settings with variable acquisition conditions [18].

On the other hand, model explainability is largely overlooked. Current caries detection systems typically operate as “black boxes”, providing classification outputs without explanatory evidence that clinicians can evaluate. This lack of transparency represents a major barrier to clinical adoption, as dentists require an understandable rationale to integrate AI findings into treatment planning and patient communication. While several studies have incorporated class activation mapping (CAM) techniques [12,19], the systematic evaluation and selection of appropriate explainability methods for dental applications remains limited.

Moreover, data augmentation strategies are not tailored to the unique characteristics of dental images. Conventional augmentation methods apply the same transformations uniformly across all images, regardless of their information content [20]. However, dental images for different types vary substantially in complexity—some contain clear diagnostic features, while others are dominated by artifacts or uniform regions. Applying the same augmentation intensity to all images may either inadequately enhance challenging cases or excessively distort already informative ones.

To address these limitations, this study proposes a comprehensive framework with three integrated components:

(1) HybridAugment+—An entropy-guided, modality-aware augmentation strategy that adaptively applies stronger augmentation to low-information images (e.g., those with specular reflections or low contrast) while preserving the integrity of already informative samples.
(2) DBAttNet—A dual-branch attention-augmented network with modality-specific attention modules. The network comprises two parallel branches: the first branch for intra-oral photographs and the second branch for radiographs. Both branches share a global channel to capture inter-channel dependencies and emphasize salient lesion regions.
(3) Caries explainability benchmarking framework—An evaluative pipeline utilizing five state-of-the-art explainability methods to optimize interpretability for dental imaging. By selecting the most robust explainer for each image modality, the framework provides high-fidelity visual evidence, ensuring that the model’s focus is anchored on salient dental lesions.

The proposed framework is evaluated on two datasets constructed from publicly available sources, comprising intra-oral photographs and radiographs, annotated by two dentists with established inter-rater reliability. Through comprehensive experiments including ablation studies, a comparison with state-of-the-art architectures, and a systematic evaluation of explainability methods, the proposed framework is demonstrated to effectively address the modality-specific challenges in caries classification while providing clinically interpretable results.

## 2. Related Work

This section reviews the existing literature on deep learning-based caries detection, organized around three themes that correspond to the challenges identified in the Introduction: (1) modality-specific approaches and their limitations, (2) data augmentation strategies in dental image analysis, and (3) explainability methods for medical image classification. For each theme, we summarize key contributions, identify remaining gaps, and position our work relative to the state of the art.

### 2.1. Deep Learning for Caries Classification

The application of deep learning to caries classification has developed rapidly over the past decade, with convolutional neural networks (CNNs) emerging as the predominant architecture. Early studies demonstrated the feasibility of transfer learning using pre-trained networks on relatively small datasets. Miki et al. [11] applied AlexNet to cone-beam CT images, achieving 88.8% accuracy on a dataset of 52 images. While this work established a proof of concept, the limited dataset size and single-center design raised questions about generalizability.

Subsequent studies employed larger datasets and more sophisticated architectures. Srivastava et al. [12] used a fully convolutional network on 3000 bitewing radiographs, reporting recall of 80.5% but precision of only 61.5%, highlighting the challenge of distinguishing early caries from artifacts. Lee et al. [21] achieved 81.0% precision using an improved VGG-19 network on 1068 radiographs, although the performance was degraded on non-ideal images typical of general practice. Kühnisch et al. [13] reported an AUC of 96.4% on 2417 oral photographs using MobileNetV2, with sensitivity of 89.6% and specificity of 94.3%, demonstrating the potential of lightweight architectures for clinical deployment.

More recent work has incorporated attention mechanisms and multi-scale feature extraction to address specific challenges. Imak et al. [17] proposed MI-DCNNE, a multi-input deep CNN, achieving 99.13% accuracy on 340 radiographs, although the small dataset and potential overfitting warrant caution. Schwendicke et al. [22] evaluated ResNet18 and ResNet50 on 226 near-infrared transillumination images, achieving an AUC of 74%, sensitivity of 59%, and specificity of 76%, underscoring the difficulty of early lesion detection. Vinayahalingam et al. [23] applied MobileNetV2 to 400 panoramic radiographs, reporting accuracy of 87% and an AUC of 90%. Moran et al. [24] compared Inception and ResNet architectures on 112 bitewing radiographs, achieving accuracy of 73.3%. Chen et al. [19] incorporated DCGAN-based augmentation with Grad-CAM explainability on 175 radiographs, achieving an F1-score of 91.39%.

In summary, most studies focus on a single imaging modality, with only a few attempting to address both photographs and radiographs within a unified framework. Furthermore, explainability remains underutilized, with only a few studies incorporating any form of model interpretation, and none provide a systematic evaluation of multiple explainability methods.

### 2.2. Data Augmentation in Medical Image Analysis

Data augmentation is a standard technique to improve model generalization by artificially expanding training datasets through transformed versions of existing images [25]. In medical imaging, augmentation helps to address the challenge of limited labeled data and introduces invariance to acquisition variations. Common augmentation operations include geometric transformations (rotation, translation, scaling), color adjustments (brightness, contrast, saturation), and noise injection [26].

Recent advanced technologies have moved beyond fixed augmentation pipelines toward adaptive and automated strategies. RandAugment [27] simplifies the augmentation search process by randomly sampling operations with a fixed magnitude, eliminating the costly search stage required by earlier methods like AutoAugment [28]. Meanwhile, Cubuk et al. [27] demonstrated that RandAugment achieves comparable or better performance regarding learned augmentation policies across multiple datasets while being significantly simpler to implement.

In dental imaging, augmentation has been applied to address modality-specific challenges. Chen et al. [19] used DCGAN-based augmentation to generate synthetic radiographs, improving the classification performance on a small dataset. Hastie et al. [29] evaluated the impact of viewing conditions on diagnostic performance, highlighting the importance of augmentation strategies that account for clinical acquisition variability. However, existing augmentation approaches for dental images apply the same transformations uniformly across all images, regardless of their information content.

### 2.3. Attention Mechanisms in Medical Image Analysis

Attention mechanisms have emerged as powerful tools to enhance CNN performance by enabling networks to focus on relevant image regions while suppressing irrelevant information [30]. In medical imaging, attention has been applied to various tasks, including lesion detection, segmentation, and classification. The convolutional block attention module (CBAM) [31] introduces a lightweight general-purpose attention module that sequentially applies channel and spatial attention, demonstrating consistent improvements across multiple architectures and datasets. Woo et al. [31] showed that CBAM learns to emphasize informative features while suppressing noise, with minimal computational overhead.

Subsequent work has developed task-specific attention mechanisms. For image denoising, Deng and Hu [32] proposed MSDNet, a multi-scale CNN with pixel-wise attention that preserves fine details while removing noise. Wu et al. [33] introduced DCANet, a dual CNN with spatial-channel attention for blind denoising, demonstrating robustness to varying noise distributions. For medical image classification, Dutta et al. [34] proposed ARM-Net, an attention-guided residual multi-scale CNN that captures multi-scale feature representations through residual connections and a lightweight global attention module. In dental imaging, attention mechanisms have seen limited application. Most studies rely on standard CNN backbones without explicit attention to modality-specific artifacts.

### 2.4. Explainable AI in Medical Imaging

The black-box nature of deep learning models poses significant challenges for clinical adoption, where understanding the rationale behind decisions is essential for trust and verification. Explainable AI (XAI) methods aim to provide insights into model decision-making by highlighting input regions that influence predictions. Class activation mapping (CAM) methods are among the most widely used XAI techniques for CNNs. Gradient-weighted class activation mapping (Grad-CAM) [35] uses gradients of the target class flowing into the final convolutional layer to produce a coarse localization map, highlighting regions that are important for prediction. Grad-CAM++ [36] extends this approach by improving gradient calculation for multiple instances of the same class. Ablation-CAM [37] introduces a gradient-free approach that generates maps by iteratively ablating feature maps, producing noise-free visualizations. LayerCAM [38] generates reliable maps from multiple CNN layers, capturing both coarse and fine object details. XGrad-CAM [39] formalizes axiomatic properties for CAM methods, demonstrating improved class discriminability through axiom-guided gradient weighting.

Explainability remains an under-addressed challenge in dental imaging. The current literature, such as the work of Chen et al. [19], primarily relies on Grad-CAM for visualizing model focus, while other studies, like that of Imak et al. [17], offer only superficial details regarding implementation. Crucially, the field lacks both a systematic comparison of CAM methods for caries detection and established quantitative metrics to evaluate the explanation quality.

Based on the above review, current studies exhibit shortcomings such as poor cross-modal generalization, a lack of specialized mechanisms to address modality-specific artifacts, and insufficient quantitative explainability. To address these issues, this study proposes a framework comprising an entropy-based adaptive augmentation strategy, a dual-branch architecture with an attention mechanism, and a CAM-based explainability method chosen through the evaluation pipeline.

## 3. Methodology

This section describes the proposed framework for dental caries classification, organized into three subsections corresponding to the three core contributions identified in the Introduction. Section 3.1 presents HybridAugment+, an entropy-guided adaptive augmentation strategy tailored to the specific challenges of intra-oral photographs and radiographs. Section 3.2 describes DBAttNet, the dual-branch attention-augmented network with modality-specific modules (IRAA for photographs, CFA for radiographs) and global refinement. Section 3.3 introduces the CAM-based interpretability framework and the quantitative metrics used to evaluate explanation quality.

### 3.1. HybridAugment+: An Entropy-Guided Adaptive Augmentation Method

Dental caries classification faces two practical imaging challenges that motivate the design of HybridAugment+: (1) in intra-oral photographs, specular reflections and non-uniform lighting frequently obscure surface lesions; (2) in radiographs, limited contrast between enamel and lesions reduces sensitivity to shallow demineralization. These modality-specific limitations require an augmentation strategy that (a) reduces lighting and color biases in photographs, (b) adaptively increases the training variability for low-information images to improve generalization, and (c) applies transformations appropriate to each modality.

#### 3.1.1. Design Rationale

In contrast to imaging technologies, which capture colors that change with lighting conditions, the human visual system maintains approximate color constancy despite changing illumination. Color balancing algorithms (e.g., Gray-World, White-Balance, Retinex) can partially restore consistency; for dental photography, the Gray-World method is attractive due to its simplicity and low computational cost [29]. Data augmentation enlarges effective training sets and improves generalization; among modern methods, RandAugment eliminates the costly search stage while providing randomized augmentation strength. HybridAugment+ combines these ideas with entropy-guided adaptation, ensuring that images with limited information content receive stronger augmentation while already informative images are preserved.

The complete HybridAugment+ procedure is formalized in Algorithm 1.

**Algorithm 1** HybridAugment+**Require**: Image I(RGB photograph or grayscale radiograph), parameters $N_{min}$ = 2, $N_{max}$ = 10, σ = 2, M = 9, Minimal constant ε
**Ensure**: Augmented image $I_{aug}$
  /* Step 1: Contrast enhancement */
  1: **if** I is RGB photograph **then**  2:  $I_{gray}=(I_{R}+I_{G}+I_{B})/3$                  /* Convert to grayscale */  3: **else**  4:  $I_{gray}$= I                       /* Radiograph already grayscale */  5: **end if**  6: $I_{eq}\;=\;CLAHE(I_{gray},\;clip\_limit=2.0,\;grid\_size\;=(8,8))$         /* Local contrast enhancement */                                                                     0/* Step 2: Color balancing (photographs only) */  7: $H_{max}=\left\lceil \log_{2}max(I_{eq})+1)\right\rceil$                 /*Identify bit-depth */  8: $I_{max\;}=2^{H_{max}}\;-1$
  9: **if** I is RGB photograph **then**10:  $I_{balanced}=\;GrayWorldColorBalance(I)$            /*Reduce illumination bias */11:  $G=(I_{eq}+\varepsilon )⊘(I_{gray}\;+\varepsilon )$                 /*Compute pixel-wise gain */12:  $I_{base\;}=\;clip(I_{balanced}\;⊗G,0,I_{max\;})$             /*Apply gain to all channels */13: **else**14:  $I_{base\;}=I_{eq}$
15: **end if**
                                                                                      0/* Step 3: Entropy computation */
16: Compute histogram $P(g_{i})$ from $I_{eq}$ for i = 0, …, $2^{H_{max}}$ – 117: $H\;=-\sum_{i=0}^{2^{H_{max}}-1}P(g_{i})\log_{2}P(g_{i})\;$               /*Shannon entropy in bits */18: $H_{norm\;}=H/H_{max}$                     /*Normalize by maximum entropy */                                                                     /* Step 4: Adaptive operation count selection */19: $p=\;N_{min}+(N_{max}-N_{min})\times \;(1-H_{norm})$             /*Expected operation count */20: $N_{sample\;}=random.normal(mean=p,std\;=\;\sigma )$           /*Stochastic sampling */21: $N_{final\;}=\;clip(round(N_{sample\;}),\;N_{min},\;N_{max})$             /*Discrete operation count*/                                                           /* Step 5: RandAugment with modality-specific operations */22: $I_{aug}=RandAugment(I_{base},\;N=N_{final},\;M=M,\;operation\_pool=Pool(I))$
/*Pool selected based on modality/*
                                                                     /*Step 6: Color preservation (photographs only) */
23: **if** I is RGB photograph **then**24:  $I_{aug}\;=\;ColorPreservation(I_{aug},\;I_{base\;})$            /*Mild gamut mapping/*25: **end if**26: **return** $I_{aug}$


#### 3.1.2. Key Components

(1) Entropy-Guided Information Assessment

To assess the complexity of the input, HybridAugment+ first computes the Contrast-Limited Adaptive Histogram Equalization (CLAHE) version of the image, denoted as $I_{eq}$. For RGB images, CLAHE is applied to the luminance channel to prevent color shifting. The Shannon entropy H is then calculated to measure the information content:

(1) $$H=-\sum_{0}^{2^{H_{max}}-1}P(g_{i})\log_{2}P(g_{i})$$

where $P(g_{i})$ is the probability of grayscale level $g_{i}$ in $I_{eq}$. Unlike standard 8-bit implementations, the maximum entropy is dynamic so as to accommodate high-bit-depth medical radiographs (e.g., 12-bit or 16-bit DICOM data). Low normalized entropy $H_{norm}$ indicates low-information images (e.g., those dominated by specular reflections or uniform backgrounds) that benefit from higher augmentation variance.

(2) Luminance-Preserving Color Balancing

For RGB photographs, simply applying contrast enhancement often leads to gamut clipping or chromatic distortion. HybridAugment+ employs a luminance injection strategy. After performing Gray-World color balancing on the original image, a pixel-wise gain factor is computed:

(2) $$G=(I_{eq}+\epsilon )⊘(I_{gray}+\epsilon )$$

The final base image $I_{base}$ is derived through $I_{base}=clip(I_{balanced}⊗G)$, ensuring that the local contrast improvements from CLAHE are seamlessly integrated into the color-balanced photograph without losing chromatic fidelity.

(3) Adaptive Operation Count

The expected operation count p is mapped from the normalized entropy through an inverse linear function:

(3) $$p=N_{min}+(N_{max}-N_{min})\times (1-H_{norm})$$

The final discrete count $N_{final}$ is sampled from a truncated normal distribution:

(4) $$N_{final}=clip(round\left(N(p,\sigma )\right),N_{min},N_{max})$$

where $N_{min}=2$, $N_{max}=10$, and σ=2 were determined empirically through validation experiments. $N_{min}=2$ ensures at least minimal augmentation for all images, while $N_{max}$ prevents excessive distortion. σ=2 provides sufficient stochasticity while maintaining a correlation with entropy-based expectations.

(4) Modality-Specific Operation Pools

The RandAugment operation pool Pool(I) is customized for each modality. For radiographs, geometric transforms and sharpness adjustments dominate, while, for photographs, additional color-space jittering is included to simulate various dental camera sensor responses, as detailed in Table 1.

The augmentation magnitude M was set to 9 on a 0–30 scale based on the validation performance, balancing sufficient transformation strength with the preservation of diagnostic features.

### 3.2. DBAttNet: Dual-Branch and Attention-Augmented Convolutional Neural Network

**Figure 1:**
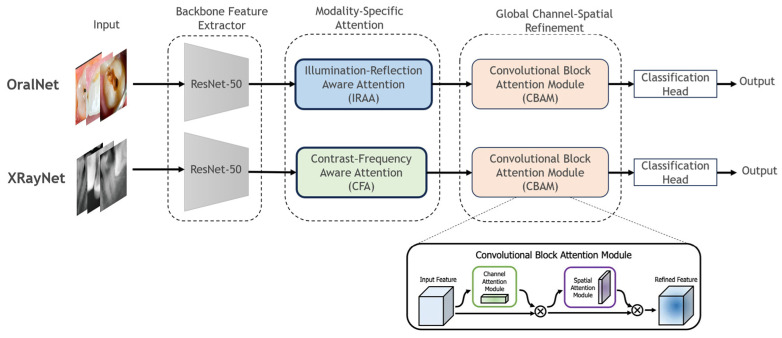
Overall architecture of the proposed DBAttNet, comprising two parallel branches (OralNet for intra-oral photographs and XRayNet for radiographs), each incorporating modality-specific attention modules (IRAA and CFA) followed by a shared CBAM for global channel–spatial refinement.

To address the heterogeneous imaging characteristics and artifact distributions between intra-oral photographs and dental radiographs, dbattnet—a dual-branch attention-augmented convolutional neural network—is proposed. Specifically, DBAttNet comprises two parallel branches: OralNet for intra-oral photographic images and XRayNet for bitewing or periapical radiographs. Figure 1 illustrates the complete architecture.

#### 3.2.1. Overall Architecture

In DBAttNet, each branch follows a four-stage design.

(1) Backbone Feature Extractor

The deep convolutional backbone ResNet50 is pretrained on ImageNet and fine-tuned for dental imagery. The backbone encodes the input image $I\in R^{H\times W\times 3}$ into a multi-scale feature representation $F\in R^{h\times w\times C}$.

(2) Modality-Specific Attention Module

Positioned immediately after the backbone, this module encodes prior knowledge of modality-specific artefacts:

OralNet employs illumination–reflection-aware attention (IRAA) to suppress high-intensity specular reflections and uneven illumination;

XRayNet employs contrast–frequency-aware attention (CFA) to enhance mid-frequency structural cues while suppressing anatomical noise.

(3) Global Channel–Spatial Refinement

Following the modality-specific stage, both branches apply CBAM to capture global inter-channel dependencies and emphasize salient lesion regions.

(4) Classification Head

The refined feature map $F^{\prime }$ is globally average-pooled and passed through a fully connected layer with Softmax activation to yield a probability vector $p\in R^{K}$ for K lesion classes:

(5) $$p=Softmax(W_{c}\cdot GAP\left(F^{\prime }\right)+b_{c})$$

where $W_{c}$ and $b_{c}$ are learnable parameters, and GAP denotes global average pooling.

#### 3.2.2. Illumination–Reflection-Aware Attention (IRAA)

Intra-oral photographs are frequently degraded by specular reflections—high-intensity highlights caused by saliva or moisture on tooth surfaces—that can obscure or mimic early caries lesions. IRAA addresses this challenge by explicitly modeling reflection regions and suppressing their contribution to the feature representation.

(1) Reflection Mask Generation

The reflection probability mask $M_{r}\in {\left[0,1\right]}^{H\times w}$ is generated during preprocessing using a heuristic approach based on intensity and saturation thresholds. Pixels with intensity > 220 (on 0–255 scale) and saturation < 30 (on 0–255 HSV scale) are identified as candidate specular reflections. These candidates are refined using morphological closing (kernel size 5 × 5) to fill small gaps, followed by Gaussian smoothing (σ=3) to produce probability values within [0, 1]. The mask generation process is not differentiable and is performed offline; the resulting mask is loaded alongside the image during training.

(2) Attention Computation

Let $F\in R^{h\times w\times d}$ denote the feature map from the backbone. IRAA computes a joint attention map $A_{IRAA}$ by integrating the global channel context and spatial reflection suppression:

(6) $$g=\sigma (W_{g}\cdot GAP\left(F\right))\in R^{d}$$

(7) $$s=1-\psi (M_{r})\in R^{h\times w}$$

(8) $$A_{IRAA}(h,w,c)=g_{c}\cdot s_{h,w}$$

where σ(·) is sigmoid activation, $GAP\left(\cdot \right)$ denotes global average pooling, $W_{g}$ is a learnable projection, and ψ(·) is bilinear upsampling to match the feature resolution. The refined feature map is obtained via

(9) $$F^{\prime }=F⊙A_{IRAA}$$

where ⊙ denotes element-wise multiplication. This formulation ensures that channels containing diagnostically relevant features are emphasized globally through g, while spatial locations corresponding to reflections are suppressed through s. The reflection mask introduces a structural bias that discourages over-attention to photometric outliers, a property not guaranteed by generic attention mechanisms.

#### 3.2.3. Contrast–Frequency-Aware Attention (CFA)

Dental radiographs suffer from low inherent contrast between enamel and dentin, compounded by anatomical noise from overlapping structures. Early demineralization is characterized by subtle textural changes and mid-frequency edge patterns that are easily masked in the spatial domain. CFA addresses this challenge by operating jointly in the spatial and frequency domains.

(1) Frequency-Domain Enhancement

Given input feature map $F\in R^{h\times w\times d}$, CFA first applies a 2D discrete Fourier transform (DFT) channel-wise:

(10) $$\hat{F}(\mu ,\nu ,c)=\sum_{x=0}^{h-1}\sum_{y=0}^{w-1}F(x,y,c)e^{-j2\pi (\frac{\mu x}{h}+\frac{vy}{w})}$$

A learnable band-pass filter B(μ,v) emphasizes mid-frequency components corresponding to edge and texture information while suppressing the low-frequency background and high-frequency noise:

(11) $$B(\mu ,v)=\sigma (\frac{\sqrt{\mu^{2}+v^{2}}-f_{l}}{\tau })\cdot (1-\sigma (\frac{\sqrt{\mu^{2}+v^{2}}-f_{h}}{\tau }))$$

where (μ,v) are centered frequency coordinates, σ(·) is the sigmoid function providing smooth transitions, $f_{l}$ and $f_{h}$ are learnable cutoff frequencies initialized as $f_{l}=0.1\times f_{max}$ and $f_{h}=0.3\times f_{max}$ ($f_{max}$ is the Nyquist frequency), and $\tau =0.05\times f_{max}$ controls the transition sharpness. The filtered frequency representation is

(12) $${\hat{F}}^{\prime }=\hat{F}⊙B$$

where ⊙ denotes element-wise multiplication (broadcast across channels). An inverse DFT restores the spatial representation $F_{freq}$. The FFT operation is performed on each channel independently, with padding to handle non-square feature maps. The computational overhead is approximately 15% compared to standard convolution.

(2) Local Contrast Attention

In parallel with frequency-domain processing, a local contrast attention map $A_{contrast}$ is computed from the spatial feature map:

(13) $$A_{contrast}(h,w)=\frac{|F(h,w)-u_{N\left(h,w\right)}|}{\sigma_{N\left(h,w\right)}+\varepsilon }$$

where $N\left(h,w\right)$ is a k×k neighborhood (empirically set to k=7), u and σ are the local mean and standard deviation, and $\varepsilon =10^{-5}$ prevents division by zero. This map highlights regions where the local intensity deviates significantly from the neighborhood average, corresponding to potential lesion boundaries.

(3) Attention Fusion

The frequency-enhanced feature $F_{freq}$ is first aggregated across channels via global average pooling (or a 1×1 convolution) to produce a spatial energy map $M_{freq}\in R^{h\times w}$. The final CFA attention map is a convex combination of normalized frequency-enhanced and contrast-based maps:

(14) $$A_{CFA}=\alpha \cdot Norm\left(F_{freq}\right)+(1-\alpha )\cdot Norm\left(A_{contrast}\right)$$

where α∈[0, 1] is a learnable parameter initialized to 0.5, and Norm(·) denotes min–max normalization to [0, 1]. The refined output is

(15) $$F^{\prime }=F⊙A_{CFA}$$

This formulation allows the network to adaptively balance frequency-domain enhancement and spatial contrast information based on the characteristics of each input image.

#### 3.2.4. Convolutional Block Attention Module (CBAM)

Following modality-specific attention, both branches apply CBAM for global channel–spatial refinement. CBAM sequentially applies the following.

(1) Channel Attention

Global average and max pooling are applied to aggregate spatial information, followed by a shared multi-layer perceptron to produce channel attention weights:

(16) $$M_{c}(F)=\sigma (MLP\left(AvgPool\left(F\right)\right)+MLP(MaxPool(F)))$$

(2) Spatial Attention

Channel-refined features are pooled along the channel dimension using average and max operations, concatenated, and passed through a convolutional layer to produce spatial attention weights:

(17) $$M_{s}(F)=\sigma ({Conv}^{7\times 7}\left([AvgPool\left(F\right)\right);MLP(MaxPool\left(F\right)]))$$

The complete CBAM operation is

(18) $$F^{\prime \prime }=M_{s}(M_{c}(F^{\prime })⊙F^{\prime })⊙M_{c}(F^{\prime })⊙F^{\prime })$$

CBAM captures complementary information across channels and spatial locations, emphasizing diagnostically relevant regions while suppressing background noise.

### 3.3. CAM-Based Explainability Framework

To enhance model transparency and enable the clinical validation of model decisions, this study integrates class activation mapping (CAM) methods into the framework. Specifically, five representative CAM-based explainability methods are included.

Grad-CAM [35]: Uses gradients of the target class flowing into the final convolutional layer to produce a coarse localization map. For a target class c, the neuron importance weights are

(19) $$\alpha_{k}^{c}=\frac{1}{Z}\sum_{i}\sum_{j}\frac{\partial y^{c}}{\partial A_{ij}^{k}}$$

where $y^{c}$ is the score for class c, $A^{k}$ is the k-th feature map, and Z is the number of pixels. The final heatmap is

(20) $$L_{Grad\text{-}CAM}^{c}=ReLU(\sum_{k}\alpha_{k}^{c}A^{k})$$

Grad-CAM++ [36]: Extends Grad-CAM by incorporating higher-order gradients to better handle multiple instances of the same class in a single image.

Ablation-CAM [37]: A gradient-free approach that generates maps by iteratively ablating (zeroing out) each feature map and measuring the change in class score, producing noise-free visualizations.

LayerCAM [38]: Generates reliable maps from multiple CNN layers by combining coarse semantic information from deeper layers with fine details from shallower layers.

XGrad-CAM [39]: Formalizes axiomatic properties for CAM methods and derives gradient weighting that satisfies these axioms, demonstrating improved class discriminability.

**Figure 2:**
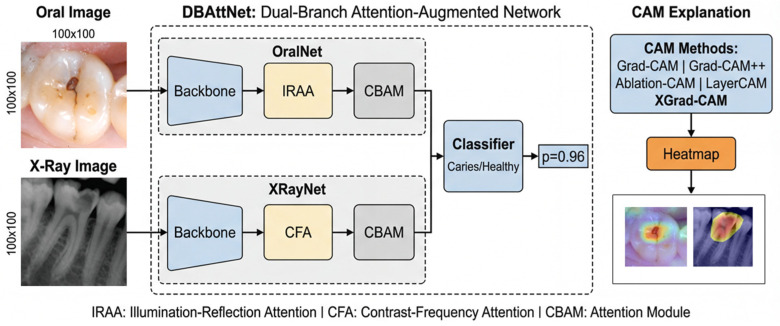
Overall framework with the proposed DBAttNet and Grad-CAM-based visual explanations.

Figure 2 provides a comparative visualization of the five CAM methods applied to DBAttNet for caries classification. The figure is organized in two rows corresponding to the two imaging modalities. The top row shows an intra-oral photograph with an occlusal caries lesion; the bottom row shows a dental radiograph with an interproximal lesion.

## 4. Experimental Setup

### 4.1. Experimental Datasets

Due to the limited availability of publicly accessible labeled caries datasets, we constructed two datasets by extracting tooth images from publicly available sources on Kaggle (https://www.kaggle.com/, accessed on 19 January 2026). The extraction process involved four steps: (1) identifying relevant dental image repositories, (2) downloading all images from the selected sources, (3) applying automated cropping to isolate individual tooth regions using a pretrained object detection model (Faster R-CNN with ResNet50 backbone), and (4) a manual quality check to exclude images with poor resolutions or severe artifacts that would preclude reliable annotation.

**Annotation Protocol**. The images were independently annotated by two licensed dentists with over five years of clinical experience in restorative dentistry. Annotation was performed at the tooth level, with each image assigned to one of three categories: caries, healthy, or non-diagnostic. For the purpose of this study, “early caries” was operationally defined as follows:

(1) For intra-oral photographs—non-cavitated lesions confined to the enamel, including white-spot lesions and initial enamel demineralization visible as opacity or discoloration without surface breakdown;
(2) For radiographs—radiolucency confined to the outer half of the enamel, without extension into the dentin.

To ensure annotation reliability, the following procedures were implemented:

(1) **Calibration**: Prior to annotation, both dentists underwent a calibration session using 50 images (25 per modality) that were not included in the final dataset. Discrepancies were discussed to establish a consensus on borderline cases.
(2) **Independent annotation**: Each dentist independently annotated all images using a standardized interface, recording both the classification and confidence level.
(3) **Inter-examiner agreement**: Agreement was assessed using Cohen’s kappa (κ) on the full dataset, yielding κ = 0.82 for photographs and κ = 0.79 for radiographs, indicating substantial agreement.
(4) **Disagreement resolution**: For cases with disagreement (approximately 8% of photographs and 11% of radiographs), the two dentists reviewed the images together and reached a consensus through discussion. Cases where a consensus could not be reached (*n* = 12) were reviewed by a third senior dentist, whose decision was final.

**Dataset Statistics**. The resulting dataset consisted of 744 intra-oral photographs and 456 radiographs. Table 2 presents the detailed distribution. For photographs, 105 images were classified as non-diagnostic (e.g., images containing restorations, severe artifacts, or multiple teeth) and excluded from the classification experiments. The class ratios (caries–healthy = 3.04:1 for photographs, 1.43:1 for radiographs) reflect the natural prevalence patterns in clinical populations and are consistent with distributions reported in previous caries detection studies [9,16].

### 4.2. Data Splitting Strategy

To ensure rigorous evaluation and prevent data leakage, we employed a two-level data splitting strategy that separated model development from the final evaluation.

First-level split—The entire dataset was randomly split into two portions:

(1) Here, 80% of the data were designated as the model development set. This portion was used for all training, validation, and model selection activities throughout the study, including feature extraction, hyperparameter tuning, architecture selection, and cross-validation.
(2) Meanwhile, 20% of the data were designated as the independent test set. This portion was immediately “frozen” and stored separately, with absolutely no access during model development. It was used only once at the end of the study to evaluate the final model’s generalization performance on unseen data.

Second-level split—On the 80% development set, we performed standard 5-fold cross-validation for model development and selection.

This design combined the best of both worlds: the 5-fold cross-validation on the development set ensured robust model selection with stability estimates, while the untouched 20% test set guaranteed an unbiased evaluation of the true generalization performance. Repeating the process 10 times further enhanced the reliability of the results by quantifying the uncertainty introduced by the random split.

### 4.3. Evaluation Metrics for Classification Verification

Because both datasets suffered from a class imbalance, a variety of metrics was used for comprehensive evaluation:

(21) $$Precision=\frac{TP}{TP+FP}$$

(22) $$Recall=\frac{TP}{TP+FN}$$

(23) $$Accuracy=\frac{TP+TN}{TP+TN+FP+FN}$$

(24) $$F1\text{-}Score=2\times \frac{Precision\times Recall}{Precision+Recall}$$

(25) $$AUC=\int_{0}^{1}TPR(t)dFPR(t)$$

(26) $$Accuracy\text{-}balanced=\frac{1}{2}\times (\frac{TP}{TP+FN}+\frac{TN}{TN+FP})$$

where TP (true positive) and TN (true negative) represent correctly classified caries and healthy tooth samples, respectively, while FP (false positive) and FN (false negative) denote misclassified samples. While accuracy can be biased toward the majority class in imbalanced settings, we additionally computed the precision–recall AUC (PR-AUC) and G-mean for comparisons.

### 4.4. Evaluation Metrics for Explainability Validation

Given the absence of pixel-level ground-truth annotations for caries lesions, this study adopted model-centric metrics as proposed in Ablation-CAM [37] to quantitatively compare the CAM methods.

Average drop (AD) measures the reduction in model confidence when the top-k% important region is masked:

(27) $$AD=\frac{1}{N}\sum_{i=1}^{N}\max\left(0,\;\frac{S_{orig}^{i}-S_{masked}^{i}}{S_{orig}^{i}}\right)\times 100\%$$

where $S_{orig}^{i}$ is the original prediction score for the target class, and $S_{masked}^{i}$ is the score after masking out the top-k% region (replacing pixels with mean values). Lower AD indicates that the removed region contributes less critically to the prediction.

Average increase (AI) measures the proportion of samples for which retaining only the top-k% important region increases the model confidence:

(28) $$AI=\frac{1}{N}\sum_{i=1}^{N}1(S_{keep}^{i}>S_{orig}^{i})\times 100\%$$

where $S_{keep}^{i}$ is the score when only the top-k% region is preserved (background masked). Higher AI suggests that the important region alone is sufficient for the prediction.

To balance the trade-off between localization fidelity (AD) and predictive sufficiency (AI), we compute a harmonic mean score:

(29) $$F_{CAM}=2\cdot \frac{\left(1-AD/100)\cdot (AI/100\right)}{\left(1-AD/100)+(AI/100\right)}$$

### 4.5. Implementation Details

All models were implemented in PyTorch 1.12 and trained on NVIDIA Tesla V100 GPUs with 32 GB memory. Common training parameters were applied across all experiments.

Optimizer: Adam with initial learning rate $3\times 10^{-4}$, $\beta_{1}=0.9$, $\beta_{2}=0.999$.

Learning rate: Cosine annealing with ${\;T}_{max}=50$, minimum learning rate $10\times 10^{-6}$.

Batch size: 16.

Epochs: 100 with early stopping.

Input size: 224 × 224 pixels (resized).

Weight decay: $10\times 10^{-4}$.

Loss function: Cross-entropy with class weights $w_{c}=N/(K\cdot N_{c})$.

## 5. Experiments and Analysis

### 5.1. Data Augmentation Experiment

To evaluate the effectiveness of the proposed HybridAugment+ strategy, a controlled comparison using four augmentation settings was conducted.

A1 (Baseline): No augmentation (baseline).

A2 (RandAugment): RandAugment with fixed parameters N=3 and M=9.

A3 (CLAHE + RandAug): CLAHE preprocessing followed by RandAugment.

A4 (HybridAugment+): Proposed entropy-guided adaptive augmentation.

Table 3 shows the comparison results of the methods on the test data.

As shown in Table 3, our proposed HybridAugment+ strategy consistently outperformed the baseline and conventional augmentation methods across all metrics. For the oral image dataset, HybridAugment+ achieved the highest performance, surpassing the baseline by margins of 5.20%, 8.46%, 6.32%, 6.83%, and 8.72%, respectively. Compared with CLAHE + RandAug (A3), HybridAugment+ provided an additional improvement of approximately 1.18% in precision, 3.07% in recall, 1.87% in accuracy, 2.12% in the F1-score, and 2.58% in the AUC, highlighting the advantage of entropy-guided, modality-aware augmentation in enhancing discriminative feature learning. A similar trend was observed in the radiograph dataset, where HybridAugment+ outperformed the other methods. Meanwhile, paired *t*-tests across folds confirmed that HybridAugment+ significantly outperformed all comparison methods (*p* < 0.05) on both datasets for all metrics.

The detailed per-fold performance for the HybridAugment+ strategy across the five folds is provided in Appendix A, Table A1, demonstrating consistent improvements over the baseline methods across all folds.

### 5.2. Classification Experiment

To evaluate the effectiveness of the proposed DBAttNet, we compared it against several state-of-the-art CNN architectures. MSDNet [32] integrates multi-scale progressive fusion and pixel-wise attention to preserve fine details in noisy images. DCANet [33] employs a dual CNN with spatial-channel attention and a noise estimation network to handle varying noise distributions. ARM-Net [34] utilizes an attention-guided residual multi-scale architecture to capture rich feature representations. Additionally, two standard baselines in medical image analysis are included, which are enhanced by the Squeeze-and-Excitation (SE) block.

Table 4 presents the comparative performance of DBAttNet against standard backbones and advanced architectures. All methods used HybridAugment+ for augmentation to ensure a fair comparison, isolating the contribution of the architectural design.

**Figure 3:**
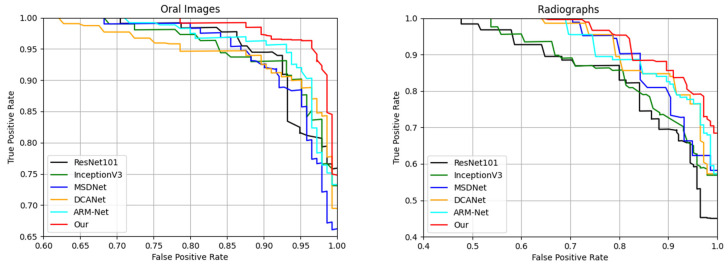
Receiver operating characteristic (ROC) curves for different models on the (**left**) oral photograph dataset and (**right**) radiograph dataset.

Table 4 presents the comparative performance of DBAttNet against standard CNN backbones (ResNet50, InceptionV3) and advanced architectures (MSDNet, DCANet, ARM-Net) on both oral photographs and radiographs. Specifically, on the oral dataset, DBAttNet achieved improvements over ARM-Net of 1.70% in precision, 0.76% in recall, 0.62% in accuracy, 1.22% in the F1-score, and 0.85% in the AUC. Notably, although ARM-Net and MSDNet already capture discriminative multi-scale features, DBAttNet further leverages its dual-stream and attention-enhanced design to achieve a superior balance between sensitivity (recall) and specificity (precision), leading to overall gains across all metrics. Similarly, on the radiograph dataset, relative to DCANet—which achieved an F1-score of 94.28%—DBAttNet provided an additional improvement of 1.48% in the F1-score and 1.44% in accuracy. The consistent advantage of DBAttNet over both ARM-Net and MSDNet further highlights its ability to adapt to the modality-specific noise distributions and structural patterns inherent in dental radiographs. Furthermore, we display the receiver operating characteristic (ROC) curves for the models in Figure 3.

Figure 3 presents the ROC curves of the different deep learning models evaluated on the two datasets: the oral dataset and radiographs. As shown, our proposed model consistently outperforms all baseline architectures across both datasets. Specifically, for the oral dataset, our method achieves a significantly higher true positive rate (TPR) across almost the entire range of false positive rates (FPRs), indicating superior classification performance. In contrast, models such as MSDNet, DCANet, and ARM-Net exhibit moderate performance, while ResNet50 and InceptionV3 demonstrate relatively lower discriminative capabilities. A similar trend is observed on the radiograph dataset, where our model again achieves the highest TPR, particularly at low FPRs, underscoring its robustness and generalization ability across imaging modalities. The notable performance under stricter classification thresholds further validates the effectiveness of our approach in complex diagnostic scenarios. The per-fold classification results for the proposed DBAttNet are reported in Appendix A, Table A2.

### 5.3. Ablation Experiment

To systematically evaluate the contribution of each architectural component in DBAttNet, an ablation study with four controlled variants was conducted.

A0 (Baseline): Single-stream ResNet50 backbone with SE blocks, no attention enhancement.

A1 (Modality-specific only): Baseline + modality-specific attention (IRAA for photographs, CFA for radiographs), no CBAM.

A2 (CBAM only): Baseline + CBAM, no modality-specific attention.

A3 (Full model): Baseline + modality-specific attention + CBAM (proposed DBAttNet).

**Figure 4:**
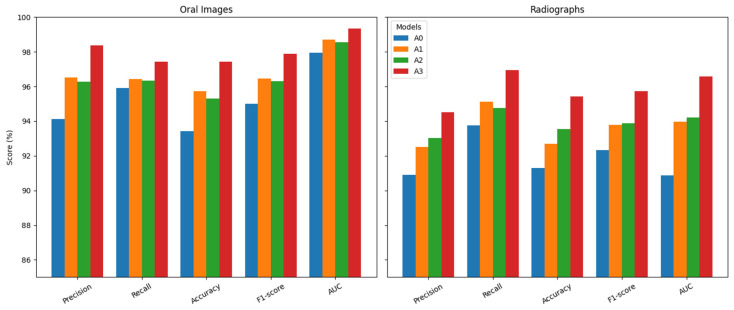
Ablation study results comparing four variants (A0: baseline; A1: modality-specific only; A2: CBAM only; A3: full DBAttNet) on the oral photograph dataset and radiograph dataset.

All variants used HybridAugment+ for augmentation and identical training parameters to ensure a fair comparison. Performance was assessed using the five metrics, and the results are presented in Figure 4.

The ablation results demonstrate that both modality-specific attention and global channel–spatial refinement substantially improve the classification performance compared to the baseline. On oral images, incorporating either IRAA (A1) or CBAM (A2) led to consistent gains over A0, while their combination (A3) achieved the best results across all metrics. A similar trend was observed on radiographs, where A3 outperformed all variants. These findings highlight the complementary effects of modality-specific and global attention, confirming their effectiveness in enhancing DBAttNet’s robustness and generalization across heterogeneous dental imaging modalities.

### 5.4. Explainability Experiment

To evaluate the suitability of different class activation mapping (CAM)-based visualization techniques for our convolutional neural network (CNN) model, we compared five representative methods: Grad-CAM, Grad-CAM++, Ablation-CAM, LayerCAM, and XGrad-CAM. All experiments were conducted using the same trained OralNet and XRayNet model with frozen weights to ensure consistency. The evaluation was performed on the two dental datasets containing only image-level labels, without pixel- or region-level ground-truth annotations. The final convolutional layer was selected as the target layer for all methods to generate class activation maps. For LayerCAM, additional multi-layer fusion was performed following the original implementation. All generated heatmaps were normalized to the range [0, 1] before further processing.

The experimental procedure for each method and each image in the test set was as follows:

(1) Generate the class activation map using the corresponding CAM technique;

(2) Identify the top-k% important region;

(3) Compute $S_{orig}^{i}$, $S_{masked}^{i}$, and $S_{keep}^{i}$;
(4) Calculate AD and AI according to the formulas above;

(5) Combine the two scores using a weighted harmonic mean inspired by the $F_{CAM}$ score.

**Figure 5:**
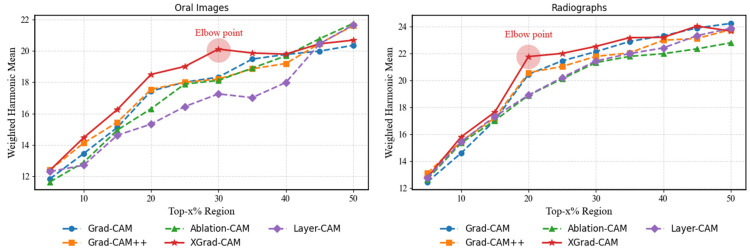
Comparative performance of five CAM methods across different top-k thresholds on the (**left**) oral photograph dataset and (**right**) radiograph dataset, evaluated using the harmonic mean of the average drop and average increase.

The comparative performance across different top-k thresholds is shown in Figure 5.

**Figure 6:**
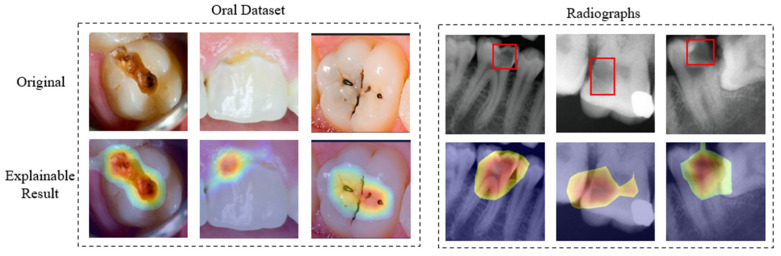
XGrad-CAM visualizations for representative samples from the (**left**) oral photograph dataset and (**right**) radiograph dataset, with the top 30% and 20% of pixels highlighted, respectively. The area within the red box indicates the approximate extent of the dental caries.

According to Figure 5, XGrad-CAM outperforms the other four CAM-based methods; we thus employed it to explain the proposed network. Furthermore, for both the oral dataset and the radiograph dataset, selecting the top 30% and 20% of pixels, respectively, achieves a balance between the average drop and average increase metrics. When the selected percentage is lower than these thresholds, the reduced explainable maps tend to omit some critical classification-related pixels, making each method more susceptible to information loss. Conversely, when the selected percentage exceeds these thresholds, the expanded explainable maps are more likely to include irrelevant or noisy pixels, potentially affecting the explainability. Moreover, Figure 6 presents the explainable visualizations for each dataset when selecting the top 30% and 20% of pixels, respectively.

For the oral dataset, the explainable maps reveal that the model successfully identifies and emphasizes the damaged regions of the teeth, confirming its ability to focus on clinically relevant features. Similarly, for the radiographs, the heatmaps highlight the areas of interest within the X-ray images, demonstrating the model’s competence in detecting dental abnormalities. Overall, the visual explanations generated using the XGrad-CAM method are derived from the network’s decision-making process (class probabilities), helping to reveal the regions that the model focuses on during prediction. This enhances the explainability of the model by providing insights into “why the model is correct” and” why it makes mistakes”, thereby facilitating its practical application.

It should be noted that this evaluation was performed in the absence of pixel-level or region-level ground-truth annotations for caries lesions. Therefore, the assessment of the CAM methods is inherently model-centric, relying on metrics such as the average drop and average increase rather than direct clinical validation. While these metrics provide quantitative insights into the faithfulness of the explanations, they do not confirm whether the highlighted regions correspond to true pathological areas.

### 5.5. Generalization Experiment

To assess the generalization capability of the proposed framework beyond dental imaging, we conducted additional experiments on publicly available medical image datasets from other domains. These experiments served two purposes: (1) evaluating whether the modality-specific attention mechanisms (IRAA and CFA) capture generalizable principles applicable to other imaging modalities and (2) comparing the performance with that of other advanced methods under identical experimental conditions.

#### 5.5.1. Experimental Design

For generalization testing, this study selected four datasets from two imaging domains that share similar challenges with dental images.

Ophthalmology faces challenges analogous to those of oral photography, as fundus photographs are subject to similar issues, including illumination variations, specular reflections, and variable acquisition conditions. To address these challenges, this study utilizes two primary retinal image datasets [40]: APTOS 2019, which contains 3662 images for diabetic retinopathy grading with varying illumination and quality, collected from multiple clinical sites using diverse acquisition protocols, and Messidor-2, comprising 1748 images characterized by variable lighting conditions and image quality, representative of real-world clinical settings.

Chest radiography encounters challenges analogous to those in dental radiographs, including low contrast, anatomical noise, and overlapping structures. For this study, two prominent datasets are selected [41]: the Kermany CXR dataset, which is a pediatric chest X-ray collection for pneumonia detection, containing 5856 images with varying degrees of pathology and normal anatomy, and the NIH ChestX-ray14 dataset, which is a large-scale repository with 112,120 images across 14 disease categories, representing diverse pathological conditions and acquisition parameters.

To ensure a fair comparison with published methods, we adopted the experimental protocols from the source papers, comparing our method against DT-Net [40], a dual-transformer network for retinal image analysis, on the APTOS 2019 and Messidor-2 datasets, and against ConvNeXt-Tiny [41], a modern CNN architecture with proven performance in chest X-ray classification, on the Kermany CXR and NIH ChestX-ray14 datasets. Our DBAttNet was adapted to each task by replacing the IRAA module (originally designed for oral photographs) with a general illumination attention module for fundus images while maintaining the same reflection suppression principles, while retaining the CFA module for chest X-rays as its contrast–frequency enhancement principles generalize to thoracic imaging, and adjusting the classification head to match the target class count for each dataset (binary for APTOS/Messidor, multi-class for NIH ChestX-ray14). All other architectural components and training procedures remained identical to those described in Section 4.5, with dataset-specific parameters (e.g., learning rate, batch size) adjusted to match the reference implementations for an equitable comparison.

#### 5.5.2. Experimental Results on Ophthalmology Datasets

Table 5 presents the comparative results on fundus image datasets. The DT-Net results are reproduced from [40] using their reported evaluation protocol.

According to Table 5, DBAttNet consistently outperformed DT-Net across both ophthalmology datasets. On Messidor-2, our method achieved 0.884 recall (vs. 0.840), 0.882 accuracy (vs. 0.860), and a 0.837 F1-score (vs. 0.790), representing relative improvements of 5.2%, 2.6%, and 5.9%, respectively. On APTOS 2019, the improvements were 2.8% in recall (0.977 vs. 0.950), 1.3% in accuracy (0.983 vs. 0.970), and 2.6% in the F1-score (0.964 vs. 0.940). The strong performance on APTOS 2019 (AUC 0.985) is particularly noteworthy given the dataset’s variability in image quality and illumination—challenges that are analogous to those addressed by IRAA in dental photographs. This suggests that the principles underlying IRAA (suppressing illumination artifacts while enhancing diagnostically relevant features) generalize effectively to other domains facing similar imaging challenges.

#### 5.5.3. Experimental Results on Chest Radiography Datasets

Table 6 presents the comparative results on the chest X-ray datasets. The ConvNeXt-Tiny results are reproduced from [41] using their reported evaluation protocol.

As shown in Table 6, on the Kermany CXR pediatric pneumonia dataset, DBAttNet achieved marginally better performance than ConvNeXt-Tiny, with its F1-score improving from 0.989 to 0.991 and the AUC from 0.988 to 0.992. While the near-ceiling baseline makes substantial gains challenging, the consistent advantage across metrics suggests that CFA’s frequency-domain enhancement provides additional discriminative information even in well-performing settings. On the more challenging NIH ChestX-ray14 dataset for 14-class multi-label classification, the improvements were more substantial, with the precision increasing from 0.703 to 0.724 (3.0% relative), recall from 0.762 to 0.776 (1.8% relative), accuracy from 0.721 to 0.742 (2.9% relative), and the F1-score from 0.731 to 0.749 (2.5% relative), although the AUC showed a slight decrease from 0.786 to 0.751—a difference that is potentially attributable to the multi-label nature, where threshold-independent metrics behave differently. The consistent F1-score improvement, which balances precision and recall, indicates that CFA’s contrast–frequency enhancement helps the model to identify subtle pathological patterns amid anatomical noise, a challenge that is directly analogous to that seen in dental radiographs.

## 6. Discussion

This study addressed three challenges in early caries detection: modality-specific artifacts, architectural adaptation, and interpretability. HybridAugment+ outperformed conventional augmentation, with its gains being more pronounced on photographs (8.72% AUC) than radiographs (7.67%), confirming that illumination-related artifacts benefit from targeted adaptive strategies. DBAttNet achieved F1-scores of 97.90% (photographs) and 95.72% (radiographs), surpassing state-of-the-art comparators. Ablation studies confirmed the synergy between modality-specific attention (IRAA/CFA) and global refinement (CBAM). The systematic CAM evaluation identified XGrad-CAM as optimal, with visualizations confirming its focus on clinically relevant regions while suppressing artifacts.

These results compare favorably with those of recent work: Kühnisch et al. [12] reported a 96.4% AUC on photographs; our approach achieved 99.02%. To our knowledge, this is the first unified framework validated on both modalities with a >95% F1-score on each, suggesting potential for supporting integrated clinical workflows pending further validation.

The generalization experiments on ophthalmology (APTOS 2019, Messidor-2) and chest X-ray datasets (Kermany CXR, NIH ChestX-ray14) demonstrated consistent performance gains over domain-specific methods (DT-Net [40], ConvNeXt-Tiny [41]), suggesting that IRAA’s illumination suppression and CFA’s contrast–frequency enhancement principles may transfer effectively to other medical imaging domains, although direct validation in these clinical contexts remains necessary.

Despite these promising results, several limitations remain: the modest dental datasets (639 photographs, 456 radiographs) from a single source limit generalizability. A further methodological limitation concerns the data splitting strategy. Due to the absence of patient-level identifiers, image-wise rather than subject-wise splitting was employed. Although a perceptual hashing step was applied to remove exact duplicates, this approach cannot fully eliminate the risk of correlated samples (e.g., multiple images from the same patient) appearing across the training and test sets. Such data leakage may lead to the overestimation of model performance and reduced generalizability to unseen patients. Therefore, the reported performance metrics should be interpreted as optimistic estimates, and external validation on independently collected datasets with patient-level separation remains essential for assessing true clinical generalizability.

Furthermore, the CAM-based explainability assessment in this study was limited by the absence of pixel-level ground-truth annotations for caries lesions. Although model-centric metrics (e.g., average drop and average increase) provide quantitative measures of explanation fidelity, they do not validate whether the highlighted regions correspond to clinically relevant lesion areas. As such, the interpretability findings should be interpreted with caution. Future work should incorporate clinical reader studies, wherein expert clinicians evaluate the plausibility and utility of the generated heatmaps, as well as annotation-based validation using expert-identified lesion boundaries where feasible. This would enable a more robust assessment of clinical interpretability.

While the cross-domain validation on ophthalmology and chest radiography datasets confirms that the framework’s core design principles transfer effectively, direct external validation on dental images from diverse institutions is still essential. Additional limitations include binary classification (vs. severity staging), heuristic reflection masks (vs. end-to-end learning), and model-centric explainability metrics lacking pixel-level ground truths. Therefore, the results presented herein should be interpreted as preliminary findings that demonstrate technical feasibility, rather than definitive evidence of clinical readiness. Future work requires multi-institutional datasets, external validation, multi-class extension, end-to-end attention, clinical reader studies, and leveraging successful cross-domain transfer to reduce the annotation burden.

## 7. Conclusions

This study proposed a framework for dental caries detection integrating three components: HybridAugment+ (entropy-guided adaptive augmentation), DBAttNet (dual-stream attention network with IRAA for photographs and CFA for radiographs), and systematic CAM-based explainability evaluation. Experiments on 639 intra-oral photographs and 456 radiographs yielded three main findings.

First, HybridAugment+ improved the performance over conventional augmentation by up to 8.72% on photographs and 7.67% on radiographs. Second, DBAttNet achieved F1-scores of 97.90% (photographs) and 95.72% (radiographs), outperforming ResNet50, InceptionV3, MSDNet, DCANet, and ARM-Net. Ablation studies confirmed the complementary contributions of modality-specific attention and global refinement. Third, a systematic comparison of five CAM methods identified XGrad-CAM as the most suitable, with optimal thresholds of 30% (photographs) and 20% (radiographs). Visual analysis confirmed the model’s focus on clinically relevant regions while suppressing artifacts.

Beyond dental validation, generalization experiments on ophthalmology (APTOS 2019, Messidor-2) and chest radiography (Kermany CXR, NIH ChestX-ray14) datasets demonstrated that DBAttNet matched or exceeded domain-specific methods (DT-Net, ConvNeXt-Tiny). These results suggest that the core design principles may generalize to other modalities facing similar imaging challenges, although further validation on modality-specific datasets is required.

## Figures and Tables

**Table 1:** The augmentation operation pool.

Image Type	Operation Pool
Oral Photographs	Brightness, contrast, gamma correction (simulate illumination)
	CLAHE/histogram perturbation (local contrast)
	Color jitter (hue/saturation small shifts)
	Small rotation (±5–15°), translation, scale
	Sharpness/unsharp mask
	Motion blur (light)
	Cutout/random occlusion (simulate specular patch)
	Additive Gaussian noise
	Local contrast adjustments (CLAHE/histogram)
Radiographs	Linear contrast/brightness
	Small rotation, translation, scale
	Gaussian noise, speckle noise
	Blurring/sharpening (mimic acquisition variability)
	Elastic deformation (mild)

**Table 2:** The information of the two datasets.

Dataset	Total	Caries	Healthy	Non-Diagnostic
Oral Images	639	481	158	105
Radiographs	456	268	188	0

Note: All data used in this study lacked patient identifiers, preventing patient-level analysis. To minimize potential data leakage from duplicate images, we performed a de-duplication step based on perceptual hashing prior to analysis. Consequently, all images were treated as independent samples. This introduces a notable limitation: without patient-level identifiers, images from the same patient may appear across training and test sets, causing data leakage that artificially inflates performance estimates. Although perceptual hashing reduces the risk of duplicate images, it cannot fully address correlations among samples from the same individual (e.g., multiple images of the same or adjacent teeth). This may lead to overly optimistic estimates of model generalizability. Therefore, the results should be interpreted cautiously, and future work should prioritize datasets with patient-level metadata to enable rigorous subject-wise splitting.

**Table 3:** Performance comparison of different augmentation strategies.

Dataset	Method	Precision	Recall	Accuracy	F1-Score	AUC	Accuracy—Balanced
Oral Images	A1: Baseline	88.92 ± 2.31	87.45 ± 2.87	87.11 ± 2.54	88.18 ± 2.42	89.23 ± 2.11	88.12 ± 2.45
	A2: RandAug	92.05 ± 1.87	91.88 ± 2.12	90.72 ± 1.96	91.96 ± 1.78	94.89 ± 2.03	91.80 ± 2.13
	A3: CLAHE + RandAug	92.94 ± 1.65	92.84 ± 1.98	91.56 ± 1.76	92.89 ± 1.54	95.37 ± 1.84	92.12 ± 2.02
	**A4: HybridAug+ (ours)**	**94.12** ± **1.43**	**95.91** ± **1.56**	**93.43** ± **1.38**	**95.01** ± **1.45**	**97.95** ± **0.79**	**92.68**± 1.22
Radiographs	A1: Baseline	85.76 ± 2.54	86.34 ± 2.76	83.64 ± 2.89	86.05 ± 2.61	85.52 ± 2.33	83.12 ± 2.14
	A2: RandAug	87.89 ± 2.12	89.14 ± 2.34	85.97 ± 2.43	88.51 ± 2.18	87.88 ± 1.98	86.55 ± 1.98
	A3: CLAHE + RandAug	88.24 ± 1.98	90.66 ± 2.21	88.79 ± 2.02	89.43 ± 1.97	88.56 ± 1.76	89.45 ± 1.78
	**A4: HybridAug+ (ours)**	**90.89** ± **1.67**	**93.76** ± **1.72**	**91.31** ± **1.64**	**92.29** ± **1.48**	**90.87** ± **1.44**	**90.34**± 1.89

Note: Values shown in bold indicate the best performance for each corresponding metric.

**Table 4:** Classification performance comparison on two datasets.

Dataset	Metric	ResNet50	InceptionV3	MSDNet	DCANet	ARM-Net	DBAttNet
Oral Dataset	Precision	94.12 ± 1.43	94.92 ± 1.38	96.21 ± 1.12	95.69 ± 1.21	96.67 ± 1.08	**98.37** ± **0.76**
	Recall	95.91 ± 1.56	93.03 ± 1.67	96.57 ± 1.08	96.77 ± 1.14	96.68 ± 1.11	**97.44** ± **0.82**
	Accuracy	93.43 ± 1.38	92.64 ± 1.43	96.23 ± 1.02	96.56 ± 1.08	96.81 ± 0.98	**97.43** ± **0.71**
	F1-score	95.01 ± 1.45	93.96 ± 1.41	96.40 ± 1.10	96.24 ± 1.12	96.68 ± 1.03	**97.90** ± **0.31**
	AUC	97.95 ± 0.79	97.94 ± 0.94	97.76 ± 0.87	97.67 ± 0.82	98.38 ± 0.73	**99.23 ± 0.55**
	Accuracy—balanced	92.68 ± 1.22	93.34 ± 1.84	96.37 ± 1.45	96.71 ± 1.47	96.88 ± 1.38	**96.93** ± **0.92**
Radiographs	Precision	90.89 ± 1.67	90.87 ± 1.71	92.59 ± 1.32	93.24 ± 1.28	93.02 ± 1.34	**94.51** ± **1.05**
	Recall	93.76 ± 1.72	96.32 ± 1.54	94.84 ± 1.41	95.33 ± 1.36	94.54 ± 1.45	**96.93** ± **1.11**
	Accuracy	91.31 ± 1.64	92.56 ± 1.48	94.01 ± 1.23	94.60 ± 1.19	94.48 ± 1.24	**95.42** ± **0.94**
	F1-score	92.29 ± 1.48	93.50 ± 1.42	93.70 ± 1.28	94.28 ± 1.22	93.78 ± 1.31	**95.70** ± **1.01**
	AUC	90.87 ± 1.44	91.92 ± 1.25	94.70 ± 1.08	95.29 ± 1.03	95.20 ± 1.11	**96.60 ± 0.85**
	Accuracy—balanced	90.34 ± 1.89	92.58 ± 1.66	94.91 ± 1.47	95.14 ± 1.32	94.64 ± 1.31	**94.95**± **1.00**

Note: Values shown in bold indicate the best performance for each corresponding metric.

**Table 5:** Generalization performance on ophthalmology datasets.

Dataset	Method	Precision	Recall	Accuracy	F1-Score	AUC
Messidor-2	DT-Net [40]	-	0.84	0.86	0.79	-
	**DBAttNet**	**0.795**	**0.884**	**0.882**	**0.837**	**0.891**
APTOS 2019	DT-Net [40]	-	0.95	0.97	0.94	-
	**DBAttNet**	**0.952**	**0.977**	**0.983**	**0.964**	**0.985**

Note: Values shown in bold indicate the best performance for each corresponding metric.

**Table 6:** Generalization performance on chest radiography datasets.

Dataset	Method	Precision	Recall	Accuracy	F1-Score	AUC
Kermany CXR	ConvNeXt-Tiny [41]	0.99	0.988	0.984	0.989	0.988
	**DBAttNet**	**0.994**	**0.989**	**0.988**	**0.991**	**0.992**
NIH ChestX-ray14	ConvNeXt-Tiny [41]	0.703	0.762	0.721	0.731	0.786
	**DBAttNet**	**0.724**	**0.776**	**0.742**	**0.749**	**0.751**

Note: Values shown in bold indicate the best performance for each corresponding metric.

## Data Availability

The data presented in this study are available via Kaggle at https://www.kaggle.com/ (accessed on 19 January 2026) and Labarchives at https://mynotebook.labarchives.com/ (accessed on 19 January 2026). These data were derived from the following resources available in the public domain: https://www.kaggle.com/nabeel1921/datasets (accessed on 19 January 2026), https://www.kaggle.com/datasets/salmansajid05/oral-diseases (accessed on 19 January 2026), and https://mynotebook.labarchives.com/share/Vahab/MjAuOHw4NTc2Mi8xNi9UcmVlTm9kZS83NzM5OTk2MDZ8NTIuOA (accessed on 19 January 2026).

## Appendix A. Per-Fold Cross-Validation Results

To enhance the transparency and support the robustness claims regarding the proposed framework, this appendix provides detailed per-fold performance metrics. The development set was divided into five folds. All results are reported as the mean ± standard deviation across the five validation folds.

**Table A1:** Per-fold performance of HybridAugment+ (%).

Dataset	Fold	Precision	Recall	Accuracy	F1-Score	AUC	Accuracy—Balanced
Oral Images	1	93.13	94.27	92.13	93.70	97.18	91.63
	2	95.75	96.79	94.81	96.27	98.01	94.23
	3	94.69	97.41	94.91	96.03	98.91	93.70
	4	92.18	94.18	92.08	93.17	97.14	91.53
	5	94.83	96.9	93.20	95.85	98.52	92.32
	Mean ± Std	94.12 ± 1.43	95.91 ± 1.56	93.43 ± 1.38	95.01 ± 1.45	97.95 ± 0.79	92.68 ± 1.22
Radiographs	1	90.12	94.95	90.65	92.47	90.6	89.92
	2	92.78	93.22	92.19	93.00	91.78	91.32
	3	88.99	91.23	89.14	90.10	89.47	88.07
	4	90.02	93.69	91.08	91.82	89.64	89.39
	5	92.53	95.69	93.49	94.08	92.85	93.01
	Mean ± Std	90.89 ± 1.67	93.76 ± 1.72	91.31 ± 1.64	92.29 ± 1.48	90.87 ± 1.44	90.34 ± 1.89

**Table A2:** Per-fold performance of DBAttNet (%).

Dataset	Fold	Precision	Recall	Accuracy	F1-Score	AUC	Accuracy—Balanced -
Oral Dataset	1	97.67	98.51	98.14	98.09	99.78	97.71
	2	98.81	96.53	97.32	97.66	98.52	96.23
	3	98.51	97.95	97.96	98.23	99.39	97.12
	4	97.53	97.46	97.41	97.49	99.66	97.85
	5	99.33	96.75	96.32	98.02	98.8	95.74
	Mean ± Std	98.37 ± 0.76	97.44 ± 0.82	97.43 ± 0.71	97.90 ± 0.31	99.23 ± 0.55	96.93 ± 0.92
Radiographs	1	93.96	96.92	94.43	95.42	95.75	93.73
	2	95.75	97.83	95.46	96.78	97.64	94.95
	3	93.79	95.15	94.53	94.47	95.71	94.19
	4	93.52	96.84	96.39	95.15	96.79	95.97
	5	95.53	97.91	96.31	96.71	97.11	95.91
	Mean ± Std	94.51 ± 1.05	96.93 ± 1.11	95.42 ± 0.94	95.70 ± 1.01	96.60 ± 0.85	94.95 ± 1.00
